# Pilomatrixoma of the parotid region: A benign tumor mimicking metastatic cutaneous squamous cell carcinoma

**DOI:** 10.1016/j.radcr.2025.01.065

**Published:** 2025-02-01

**Authors:** Nikita Grieder, Marcella Pucci, Claudio De Vito, Yan Monnier, Nicolas Dulguerov, Maxime Mermod

**Affiliations:** a Department of Otolaryngology - Head and Neck Surgery, University Hospitals of Geneva, Geneva, Switzerland; b Department of Radiology, University Hospitals of Geneva, Geneva, Switzerland; c Department of Clinical Pathology, University Hospitals of Geneva, Geneva, Switzerland

**Keywords:** Pilomatrixoma, Squamous cell carcinoma, Fine needle aspiration biopsy

## Abstract

Head and neck tumors in adults present a broad differential diagnosis, particularly when considering malignant neoplasms that require prompt diagnosis and intervention. We report the case of a 66-year-old woman with a progressively enlarging mass in the left parotid region. Initial assessments, including fine needle aspiration biopsy and imaging studies from outside institutions, suggested a diagnosis of squamous cell carcinoma. However, final histopathological analysis revealed that the mass was consistent with a pilomatrixoma. This case highlights the critical importance of meticulous radiological interpretation and the role of fine needle aspiration cytology (FNA) in accurately distinguishing between these 2 entities before initiating treatment.

## Introduction

Pilomatrixoma is a benign skin tumor that originates from hair follicles. It typically presents as a firm, slowly growing mass with discolored overlying skin, often located in the head and neck region. This lesion is more common in the first 2 decades of life, with a slight predominance in females. Characteristic radiological features include well-defined margins, a lack of deep invasion, and calcifications within the lesion [1].

Cytology can be unreliable, with up to 45% of pilomatrixomas being misdiagnosed as other benign lesions [2]. Histological examination is crucial for accurate diagnosis, as it reveals the presence of matrical (basaloid) cells and distinctive shadow or ghost cells.

Here, we present a rare case of pilomatrixoma, which was initially misdiagnosed as metastatic cutaneous squamous cell carcinoma based on history, FNA cytology and FDG PET-CT findings.

## Case presentation

**Fig. 1:**
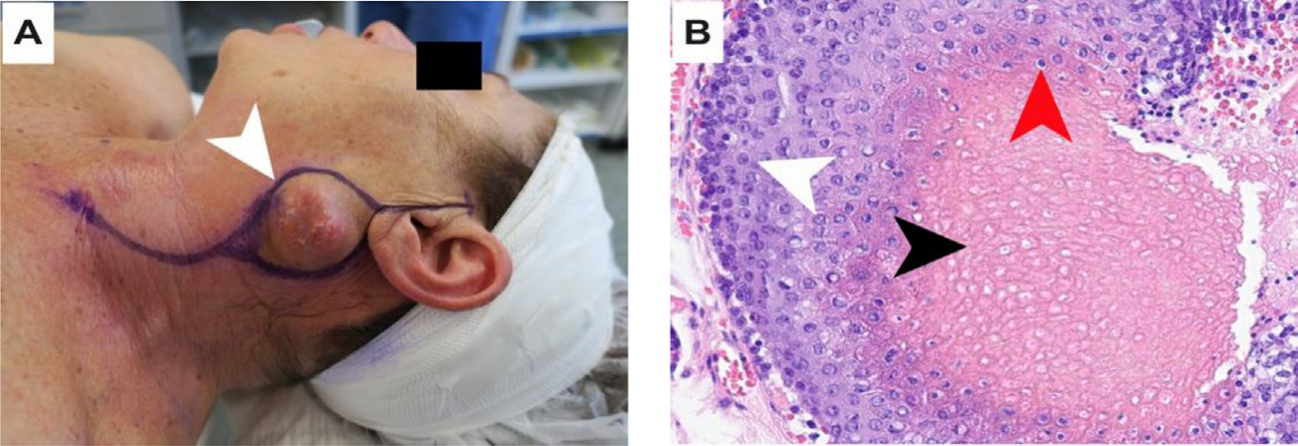
(A) Left parotid mass (white arrowhead), with the incision line in purple. (B) Histological image (40x magnification) with hematoxylin-eosin stain, presence of shadow cells (black arrowhead), intermediate cells (red arrowhead), matrical cells (white arrowhead).

A 66-year-old woman, previously in good health and with a history of UV exposure, presented with a 2-month onset of a progressively enlarging mass on the left side of her neck. On physical examination, a firm, subcutaneous mass was felt in the parotid region, near the tail (Fig. 1A). There was no evidence of pain, discharge, or cranial nerve impairment. A complete head and neck examination including nasofibroscopy was performed and did not reveal any suspicious lesion. There was no associated palpable lymphadenopathy.

Ultrasound-guided fine needle aspiration biopsy had been performed at an external institution and reported the presence of aggregates of abnormal cells with elevated nuclear-cytoplasmic ratio and the expression of the p63 marker, suggestive of poorly differentiated squamous cell carcinoma.

**Fig. 2:**
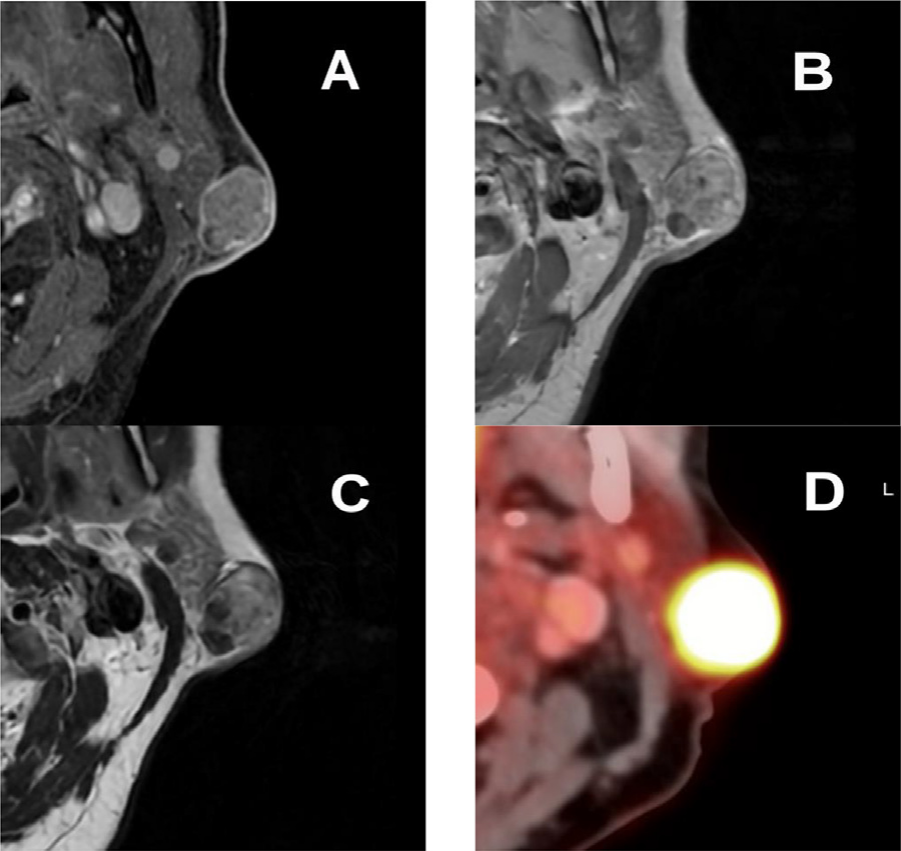
(A) Gadolinium-enhanced fat saturation T1 weighted images MRI with marked enhancement of the lesion. (B) Gadolinium-enhanced T1 weighted images MRI with heterogenous enhancement of the lesion. C) T2-weighted images MRI showing a heterogenous subcutaneous mass in contact with the parotid gland. D) FDG PET-CT showing a hypermetabolic mass in the left parotid region with a SUV of 18.

Considering the cytology findings, a FDG PET-CT was done and detected a hypermetabolic nodule at the parotid tail without further suspicious areas (Fig. 2D). The mass showed a standardized uptake value (SUV) of 18. Considering those findings, a magnetic resonance imaging (MRI) was performed to better assess local extension. The latter identified a 2.2 × 1.6 × 1.2 cm (AP x LL x CC) heterogenous subcutaneous mass in close contact with the parotid tail extending into the dermis on the T2-weighted sequence (Fig. 2C). On gadolinium-enhanced T1 weighted images, marked enhancement was observed (Fig. 2A, B).

**Fig. 3:**
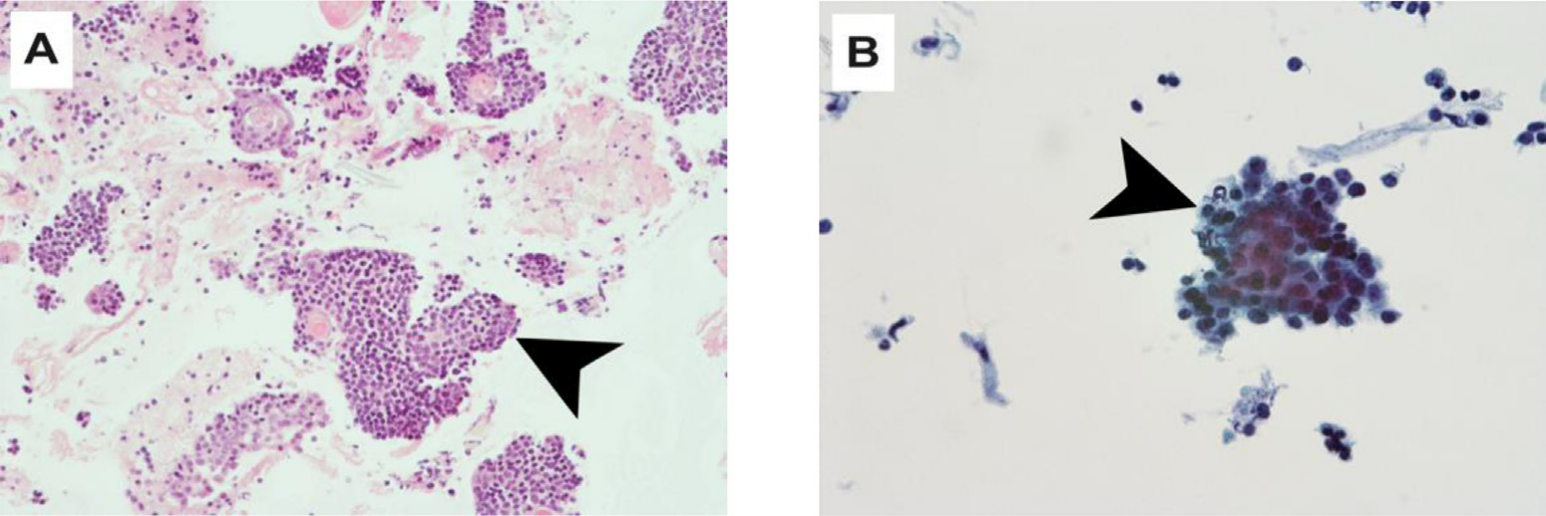
(A) Histological image (20x magnification) with hematoxylin-eosin stain, presence of basaloid cells. (B) Histological image (45x magnification) with Papanicolaou stain, presence of basaloid cells with elevated nuclear-cytoplasmic ratio.

A full dermatological assessment confirmed the absence of primary skin cancer. The case was discussed at the multidisciplinary tumor board. The outside cytology FNA slides were not reviewed at the time of the treatment (Fig. 3A and B). The diagnosis retained was metastatic cutaneous squamous cell carcinoma (cTxcN1M0) to the parotid. A therapeutic decision was made for a left parotidectomy with an ipsilateral selective neck dissection. Final histology of the resected specimen showed basaloid cells, intermediate cells and shadow cells (Fig. 1B), with few calcium deposits (<1 mm) and fibrosis, which led to the diagnosis of pilomatrixoma.

The patient underwent successful surgical excision, with no recurrence observed at the 6-month follow-up.

## Discussion

Pilomatrixoma is a benign skin tumor that originates from hair matrix cells. It was initially misclassified in 1880 as calcifying epithelioma, but this error was corrected in 1961 when it was renamed to reflect its true origin in the outer root sheath of hair follicles [3].

This neoplasm exhibits a bimodal age distribution, commonly presenting in 2 age groups: children aged 1-20 and adults aged 50-65, with a higher incidence in pediatric patients [5]. The male-to-female ratio is approximately 1:1.5, indicating a slight female predominance [6]. Pilomatrixomas typically occur on the head and neck region but can also appear on the upper limbs, trunk, and occasionally the lower limbs. Clinically, they present as firm, nontender subcutaneous nodules, usually well-defined and attached to the skin, ranging from 0.5 to 6 cm in size, with possible color changes in the overlying skin [3,4].

Diagnosing pilomatrixoma can be challenging due to the limited accuracy of fine needle aspiration biopsy, which ranges from 33% to 44% [2]. Differential diagnoses include various cysts, malignancies such as squamous cell and basal cell carcinoma, vascular anomalies, and more [[2], [3], [4]]. Histologically, pilomatrixomas are characterized by basaloid matrical cells, shadow cells, and calcification. Misinterpretation can occur if atypical basaloid cells exhibit a high nuclear-cytoplasmic ratio, mimicking malignancy [5]. Additionally, foreign body-type multinucleated giant cells and calcifications are frequently observed [6,7].

Imaging studies like MRI and FDG-PET/CT can suggest the presence of pilomatrixoma but lack specificity. MRI findings vary, showing intermediate signal intensity with possible enhancement, while FDG-PET/CT may reveal hyper-metabolic activity, which can misleadingly suggest malignancy due to inflammatory processes [8,9].

Our case serves as a reminder that FDG uptake is not cancer specific, as false-positive uptake can also occur in benign conditions such as inflammatory lesions. Moreover, other causes of nonmalignant FDG uptake include contralateral vocal cord palsy, autoimmune thyroiditis, thymus hyperplasia, lymphoid follicular hyperplasia, and specifically in the parotid, Whartin's tumor [10].

The definitive treatment is surgical resection, which leads to a low recurrence rate of 0%-1.4% [2]. Pilomatrix carcinoma, a rare malignant variant can occur, characterized by features being atypical immature matrical basaloid cells with numerous mitosis and infiltrative borders. All of these findings were not present in our patient.

Diagnostically, the presence of shadow cells with pale anucleated centers is pivotal yet challenging to detect in cytologic smears or fine needle aspiration biopsies due to detachment difficulties. Basal cells with a high nuclear-cytoplasmic ratio and prominent nucleoli may be mistakenly identified as malignant [5]. Core biopsy's accuracy was not found and doesn't seem exempt of unsatisfying material. Extemporaneous examination of the resected lesion must be the best option, but not always performed in cases where the diagnosis is not doubted.

Our case led to a selective neck dissection which would not have been indicated for this type of lesion.

## Conclusion

Pilomatrixoma is a benign skin tumor derived from the hair follicle. It should be considered in the differential diagnosis of an FDG-avid subcutaneous nodule. The range of potential diagnoses for neck masses is large, and although rare, pilomatrixoma can present with features that suggest malignancy on imaging and cytology.

Surgeons, radiologists, and cytopathologists should be aware of this entity to prevent overtreatment.

## Patient consent

Complete written informed consent was obtained from the patient for the publication of this study and accompanying images.
